# Prediction of undernutrition and identification of its influencing predictors among under-five children in Bangladesh using explainable machine learning algorithms

**DOI:** 10.1371/journal.pone.0315393

**Published:** 2024-12-06

**Authors:** Md. Merajul Islam, Nobab Md. Shoukot Jahan Kibria, Sujit Kumar, Dulal Chandra Roy, Md. Rezaul Karim

**Affiliations:** 1 Department of Statistics, Jatiya Kabi Kazi Nazrul Islam University, Trishal, Mymensingh, Bangladesh; 2 Department of Statistics, University of Rajshahi, Rajshahi, Bangladesh; Khulna University, BANGLADESH

## Abstract

**Background and objectives:**

Child undernutrition is a leading global health concern, especially in low and middle-income developing countries, including Bangladesh. Thus, the objectives of this study are to develop an appropriate model for predicting the risk of undernutrition and identify its influencing predictors among under-five children in Bangladesh using explainable machine learning algorithms.

**Materials and methods:**

This study used the latest nationally representative cross-sectional Bangladesh demographic health survey (BDHS), 2017–18 data. The Boruta technique was implemented to identify the important predictors of undernutrition, and logistic regression, artificial neural network, random forest, and extreme gradient boosting (XGB) were adopted to predict undernutrition (stunting, wasting, and underweight) risk. The models’ performance was evaluated through accuracy and area under the curve (AUC). Additionally, SHapley Additive exPlanations (SHAP) were employed to illustrate the influencing predictors of undernutrition.

**Results:**

The XGB-based model outperformed the other models, with the accuracy and AUC respectively 81.73% and 0.802 for stunting, 76.15% and 0.622 for wasting, and 79.13% and 0.712 for underweight. Moreover, the SHAP method demonstrated that the father’s education, wealth, mother’s education, BMI, birth interval, vitamin A, watching television, toilet facility, residence, and water source are the influential predictors of stunting. While, BMI, mother education, and BCG of wasting; and father education, wealth, mother education, BMI, birth interval, toilet facility, breastfeeding, birth order, and residence of underweight.

**Conclusion:**

The proposed integrating framework will be supportive as a method for selecting important predictors and predicting children who are at high risk of stunting, wasting, and underweight in Bangladesh.

## 1. Introduction

Malnutrition refers to deficiencies, excesses, or imbalances in an individual’s energy and/or nutrient intake [1]. It encompasses two main types of medical conditions. The first is "undernutrition," which includes stunting, wasting, and underweight [2]. The other is non-communicable diseases linked to unhealthy diets, such as overweight, obesity, and related problems. Malnutrition, particularly undernutrition in early childhood, has an adverse effect on children’s physical and mental development and poses a significant risk for various chronic diseases, including both communicable and non-communicable [3–5]. Undernutrition can lead to individuals becoming undernourished, making them more susceptible to illness, increasing their chances of infection, and raising the risk of fractures [6]. The nation’s economy suffers long-term consequences from this problem, which also seriously impedes advancement. It is estimated that undernutrition accounts for one-third of sickness and mortality among children aged 59 months and under, and nearly 3.5 million fatalities worldwide [7, 8]. UNICEF reports that the current population of Bangladesh is 169.8 million, with 16.3 million being children under the age of five. It has been reported that approximately 9.5 million (54%) children are stunted, 17% are wasted, and 56% are underweight [9]. Despite the decrease in rates of undernutrition over the past few decades, child undernutrition remains a significant issue for Bangladesh. To enhance the management and control of undernutrition risk among children under five, it could be beneficial to employ a smart system that utilizes modern technologies to identify undernourished children early on [10]. Early detection of the associated risk factors as well as accurate diagnosis of the risk of undernutrition can play a key role in timely intervention with implementation in preventing undernutrition and other associated diseases. Thus, early detection of undernourished children and the identification of the contributing variables to their condition requires the implementation of a smart system.

Nowadays, machine learning (ML) is a modern technology that falls under the umbrella of artificial intelligence (AI). It is designed to identify patterns within data autonomously and utilize this information to make predictions. ML-based automated models that have been developed recently are gaining more and more interest for their ability to predict the risk of malnutrition among children under the age of five in various countries [11–19]. Over the past decade, a few studies have been done to develop a predictive tool for predicting the risk of undernutrition in Bangladesh [20–22]. The development of the prediction models for undernutrition is influenced by various factors that show considerable variation across different countries or regions over time. Rahman et al. [21] applied some ML algorithms to identify the risk factors of malnutrition based on Bangladesh Demographic Health Survey (BDHS), 2014 data. They utilized logistic regression (LR) model to identify the important factors of undernutrition. This study uses the latest BDHS, 2017–18 data and considers more predictors than those included in the referenced study [21]. The Boruta approach, which is a wrapper-based feature selection approach based on random forest (RF) classifier and can handle complex non-linear data with correlated features more effectively than the LR method, is applied here for selecting the important features. The paper utilizes the popular ML-based algorithms (LR, ANN, RF) and additionally extreme gradient boosting (XGB) for comparing models’ performance. Also, determining the influencing predictors that contribute to the outcome prediction using the SHapley Additive exPlanations (SHAP) method is one of the main focuses of this study.

**Fig 1:**
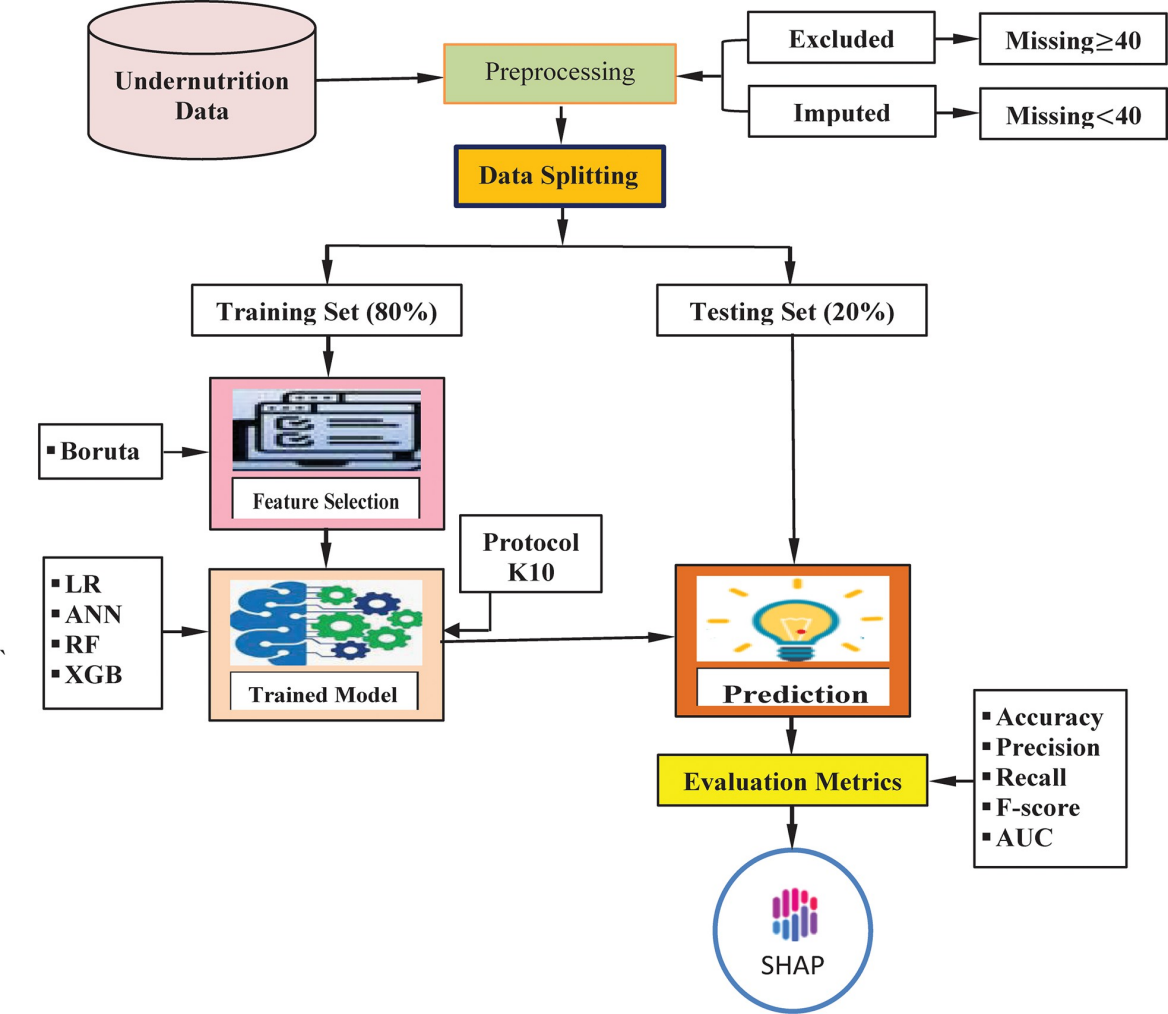
Diagrammatic representation of the proposed framework.

An attempt has been made in this study to use an explainable ML-based model for the prediction of undernutrition status among under-five children in Bangladesh. Therefore, this study’s objective was to develop an appropriate model using explainable ML algorithms that predict the risk of undermatron among under-five children in Bangladesh. Furthermore, for model interpretation, the study has successfully determined the influential predictors that contribute to the prediction of undernutrition using SHAP, which is a post hoc model interpretation technique viz. theoretically based on the Shapley value. Consequently, this information can then be used as a guide for individualized prevention and treatment to prevent the development of undernutrition among under-five children in Bangladesh. The diagrammatic representation of the proposed framework is displayed in **Fig 1**.

The organization of the remaining part of this work is structured as follows: Section 2 introduces the materials and methods utilized. The results are shown in Section 3, and a thorough discussion is given in Section 4. Finally, the conclusions are presented in Section 5.

## 2. Materials and methods

### 2.1 Data source and study design

The dataset utilized in this study was obtained from the BDHS, 2017–18. This is the most recent comprehensive survey that includes all of the enumeration areas (EAs) of the nation. The samples of this survey were collected from different households using two-stage stratified cluster sampling [23]. A total of 675 EAs were chosen in the 1^st^ stage, with the probability of selection being equal to the size of the EA. In the 2^nd^ stage, 30 households were chosen with a systematic procedure from each selected EA. About 18, 000 ever-married women were selected to participate in the interview, with 17,863 (99%) of them completing the interview successfully. In the survey conducted in 2017–18, a total of 8,759 children under the age of five were identified as eligible for anthropometric measurement. Finally, excluding refused, don’t know, flagged cases, and others (technical problems), a total of 7,796 stunting, 7,777 wasting, and 7,838 underweight cases were incorporated for analysis.

### 2.2. Ethical approval

This study made use of an available public domain survey dataset from the BDHS, 2017–18. The BDHS surveys received ethical approval from ICF Macro Institutional Review Board, Maryland, USA, and the National Research Ethics Committee of Bangladesh Medical Research Council (BMRC), Dhaka, Bangladesh, that is why it does not require any additional ethical approval.

### 2.3. Outcome variables

The outcome variable of this study was undernutrition (stunting, wasting, and underweight) among under-five children. Stunting was measured by height-for-age Z-score (HAZ), wasting weight-for-age Z-score (WAZ), and underweight weight-for-height Z-score (WHZ) [24, 25]. According to WHO, children with HAZ<-2 standard deviation (SD) were considered stunted, WAZ<-2SD were wasted, and WHZ<-2 were underweight [26]. These variables were encoded in binary form, with values of 1 and 0 (1 for stunted, 0 for not stunted), (1 for wasted, 0 for not wasted), and (1 for underweight, 0 for not underweight).

### 2.4. Predictors

This study considered different demographic, socioeconomic, behavioral, and medical-related explanatory variables as predictors for undernutrition based on the accessibility of the dataset, self-efficacy, and previous sittings [27–32]. The predictors are age, residence, region, sex, mother education, father education, working, BMI, members, religion, wealth, breastfeeding, decision contraception, twin child, child sex, total children, birth order, birth interval, age at 1^st^ birth, BCG, vitamin A, diarrhea, fever, cough, water source, cooking fuel, toilet facility, and watching television. **Table 1** represents a detailed description and categorization of the chosen predictors.

**Table 1:** Description and categorization of the predictors.

Predictors	Descriptions	Categorization
Age	Age of the respondents	15–19, 20–24, 25–29, 30–34, 35–39, 40–44, 45–49
Residence	Type of place of residence	Urban, rural
Division	Division of residence	Barisal, Chittagong, Dhaka, Khulna, Mymensingh, Rajshahi, Rangpur, Sylhet.
Sex	Sex of household head	Male, Female
Mother education	Mother education level	No education, Primary, Secondary, Higher
Father education	Husband education level	No education, Primary, Secondary, Higher
Working	Mother working status	No, Yes
BMI	Body mass index	Underweight, Normal, Overweight, Obese
Members	Household’s members	≤4, >4
Religion	Religion status	Muslim, Others
Wealth	Combined wealth status	Poorest, Poorer, Middle, Richer, Richest
Breastfeeding	Currently breastfeeding	No, Yes
Contraception	Decision maker for using contraception	Mother, husband, joint, others
Twin child	Child is twin	Single, 1^st^ of multiple, 2^nd^ of multiple
Child sex	Sex of child	Male, Female
Total children	Total children ever born	≤2, >2
Birth order	Birth order number	1, 2–3, >3
Birth interval	Preceding birth interval (months)	<24, 24–47, >47
Age at 1^st^ birth	Age of respondent at 1^st^ birth	<18, ≥18
BCG	Received Bacillus Calmette-Guerin vaccine	No, Yes
Vitamin A	Received vitamin A1	No, Yes
Diarrhea	Had diarrhea	No, Yes
Fever	Had fever	No, Yes
Cough	Had cough	No, Yes
Water source	Sources of drinking water	Improved, Unimproved
Cooking fuel	Cooking fuel status	Improved, Unimproved
Toilet facility	Toilet facility status	Hygienic, Unhygienic
Watching television	Television watching habit	No, Yes

### 2.5. Statistical analysis

The study participants’ background **characteristics were reported** as numbers (%) for the chosen predictors. Pearson’s ***χ***^**2**^ **test was executed to** examine the association between different predictors and stunting, wasting, and underweight. Statistical package SPSS and programming languages R and Python were applied to analyze the data.

#### 2.5.1. Handling missing value

Missing data is the absence of values or information that should ideally be present in a dataset. In any data collection or analysis process, missing data can occur for a wide range of reasons, including, such as technical issues, human error, non-response, and so on. Addressing missing data is an essential aspect of data analysis that has the potential to affect accuracy and reliability [33]. In this study, predictors with<40% missing values were taken into consideration, while predictors with ≥40% were eliminated from the dataset [34, 35]. There are several methods for handling missing data, including dropping missing values and filling missing values. This study employed a widely popular K-NN method to address the issue of missing values [36–38].

### 2.6. Data partition

The dataset was partitioned into two sets: training and testing, using a random partitioning method with an 8:2 ratio. There were 6237 children in the training set and 1559 in the testing set for stunting, 6222 children in the training set and 1555 in the testing set for wasting, 6270 children in the training set, and 1568 in the testing set for underweight.

### 2.7. Feature selection

Feature selection, also known as variable/attribute selection in statistics as well as ML, plays a vital role in developing an effective prediction model by choosing the most important features. It can also lead to enhanced performance of the model, better generalization, speedier computing, and greater interpretability [39]. We utilized the Boruta feature selection method in this work to identify the important predictors of stunting, wasting, and underweight in the training phase. Boruta is a wrapper-based approach that makes use of the RF classifier and outperforms others because it is consistent and unbiased [40]. The following steps were used in Boruta method to identify the important predictors

**Step 1:** Create a shadow dataset by shuffling the values of each predictor randomly.
**Step 2:** Merge the original and shadow datasets to make a single dataset.
**Step 3:** Train a RF classifier by utilizing the merged dataset and assess each predictor’s significance using a variable importance measure.
**Step 4:** Calculate the Z-score for each predictor by utilizing the predictor’s importance values. The Z-score can be determined using the following formula: Z-score = (Predictor Importance—Mean (Shadow Predictor Importance)) / Standard Deviation (Shadow Predictor Importance).
**Step 5:** Predictors exceeding a specific threshold Z-score (typically positive) are labeled as "Confirmed," while predictors falling below this threshold are labeled as "Rejected."
**Step 6:** Repeat this process until all predictors are either confirmed or rejected.

### 2.8. Machine learning algorithms

The current study adopted three distinct types of popular ML-based algorithms to predict undernutrition risk among under-five children in Bangladesh (**Table 2**).

**Table 2:** Types of machine learning algorithms.

Type	Algorithms
Classical	Logistic regression
Non-linear	Artificial neural network
Ensemble	Random forest and Extreme gradient boosting

#### 2.8.1. Logistic regression

Logistic regression (LR) is a commonly utilized statistical method in predictive modeling for predicting the outcome of a categorical dependent variable [41]. The LR method uses the sigmoid function to determine the probability of the outcome variable based on the input predictors. The logistic regression equation can be defined as follows:

$$\log_{e}\left(\frac{p_{i}}{1‐p_{i}}\right)=\alpha +\beta_{1}x_{1i}+\beta_{2}x_{2i}+\ldots ..+\beta_{k}x_{ki}+\epsilon_{i},i=1,2,\ldots ,n$$ (1)

Where, *p*_*i*_ indicates the probability of undernutrition for *i*^*th*^ children and 1−*p*_*i*_ indicates the probability of non-undernutrition; *x*_*ki*_ is the *k*^*th*^ input predictors of the *i*^*th*^ children and β_*k*_ is the *k*^*th*^ regression coefficients. The maximum likelihood method was utilized to estimate the model parameters for the logistic regression equation. Eq (1) can be represented as

$$p=\frac{exp(\alpha +\beta_{1}x_{1i}+\beta_{2}x_{2i}+\ldots ..+\beta_{k}x_{ki})}{1+\exp(\alpha +\beta_{1}x_{1i}+\beta_{2}x_{2i}+\ldots ..+\beta_{k}x_{ki})}$$ (2)

and odds as

$$\frac{p}{1-p}=exp(\alpha +\beta_{1}x_{1i}+\beta_{2}x_{2i}+\ldots ..+\beta_{k}x_{ki})$$ (3)

If $\frac{p}{1-p}\geq 1$, predict class 1 (undernutrition); otherwise, predict 0 (not-undernutrition).

#### 2.8.2 Artificial neural network

An artificial neural network (ANN) is a type of non-linear ML technique that can be utilized to perform a variety of tasks, including classification, regression, and so on [42]. It consists of interconnected nodes, called neurons, which are arranged into three layers. including an input layer, one or more hidden layers, and an output layer. During training, the network adjusted the weights and biases linked to each neuron to minimize error. This process is carried out by employing an optimization algorithm, like gradient descent, that iteratively updates the weights and biases through the sigmoid activation function. The function can be expressed as follows

$$\sigma (z)=\frac{1}{1+e^{-z}}$$ (4)

Here, *z* is the input. The procedure is repeated until the values of the iteration remain unchanged.

#### 2.8.3 Random forest

Random forest (RF), introduced by Breiman, is a versatile ensemble-based ML algorithm [43]. The RF model was constructed using the following steps

**Step 1:** Select sample data using the bootstrap method from the training set
**Step 2:** Construct a decision tree (DT) for each sample data.
**Step 3:** Build a forest with 500 trees or more by repeating **Step 1** and **Step 2**
**Step 4:** Take into account the predictions made by each formed DT, then use a majority vote to determine the final prediction.

#### 2.8.4 Extreme gradient boosting

Extreme gradient boosting (XGB) is a highly effective ensemble learning algorithm commonly used in various fields such as classification, regression, and ranking [44]. The algorithm is developed based on the principles of gradient boosting framework. It works iteratively through the use of decision trees, each aimed at correcting the errors of the previous trees. For binary classification, a logistic loss function with logistic transformation is useful for deriving the predicted probabilities from the model predictions. `The logistic loss function is defined as

$$Loss(y_{i},p_{i})=-[y_{i}\;\log\left(p_{i}\right)+(1-y_{i})log(1-p_{i})]$$ (5)

Where, *y*_*i*_ is the true class label of *i*^*th*^ children and *p*_*i*_ is the predicted probability that the *i*^*th*^ children belong to the positive class.

### 2.9. Hyperparameters tuning

Hyperparameters in machine learning are variables whose values are predefined before to the start of the learning process. They control the execution of the learning algorithm, affecting factors such as learning rate, regularization strength, and model complexity. Tuning these hyperparameters is crucial for optimizing model performance. The grid search approach with 10-fold cross-validation (CV) protocols was employed to tune the hyperparameter values in the training phase.

### 2.10. Performance evaluation metrics

The model’s performance was assessed by accuracy, precision, recall, and F-score in the testing set [45–47]. These values of the performance metrics are calculated based on the confusion matrix via four measurements: true positive (TP), false negative (FN), false positive (FN), and true negative (TN). Also, the area under the curve (AUC) is considered for the evaluation of the models. The AUC is a single value representing the area under the ROC curve, demonstrating the model’s ability to discriminate between undernutrition and non-undernutrition. It is mathematically represented as

$$AUC=\int_{x=0}^{1}TPR({FPR}^{-1}(x))dx$$ (6)

The probability curve, known as the ROC curve, shows the relationship between sensitivity and 1-specificity at various classification cut-off points. It is a widely used metric for evaluating the predictive effectiveness of machine learning models in medical diagnostics [48].

### 2.11. Predictor’s assessment using SHAP analysis

The traditional output of the XGB model only sorts the importance of variables, but it does not provide a way to assess the direction and magnitude of their impact on outcomes. SHAP is a widely used framework for interpretability in machine learning [49]. It assigns the prediction of a model to its individual features, determining how much each feature contributes to the final outcome through visualization. It is based on Shapley values derived from additive feature attribution methods, originally introduced by Lloyd Shapley in the field of game theory [50]. This approach provides a fair solution for each participant in the models by offering a wide range of features, including consistency, efficiency, dummy, and additively. The efficiency property of the SHAP method leads to more reliable outcomes when compared to alternative methods, like local interpretable model-agnostic explanations. However, predictors that have a positive SHAP value aid in the prediction of children with undernutrition in the model, while predictors with a negative SHAP value aid in the prediction of children with not undernutrition. Particularly, the importance of individual predictor, say the *k*^*th*^ predictor is ascertained through the Shapley value, which is computed using the following formula

$$\emptyset_{i}(v)=\frac{1}{M!}\sum_{S\subseteq M\setminus \{k\}}\left|S|!(M-|S|-1)![v(S\cup \{k\})-v(S)\right]$$ (7)

Where, *S* represents the subset of predictors that do not contain the predictor for which we are determining the value of ∅_*k*_(*v*); *S*∪{*k*} represents the group of predictors that includes *S* as well as the *k*^*th*^ predictor; *v*(*S*) represents the outcome of an ML-based model that utilizes the predictors from *S*. *S*⊆*M*\{*k*} means all sets of *S* in *M* predictors, excluding the *k*^*th*^ predictor.

## 3. Results

### 3.1 Background characteristics

**Table 3** represents the background characteristics of the study participants. This study reported that the overall prevalence of stunting was 31.3%, wasting 8.5%, and underweight 22.5%. The average height, weight, and age of the children were 83.07±14.60 cm, 10.77±3.41 kg, and 28.61±17.58 months, respectively, and mostly resided in rural areas. Mothers aged 45–49 years showed the highest percentage (66.7%) of stunting, whereas mothers aged 40–44 years revealed the largest percentage (10.6%) of wasting, and mothers aged 45–49 years showed the largest percentage (53.3%) of underweight. Sylhet division showed the highest percentage of being stunting (41.2%), wasting (9.9%), and underweight (30.8%) compared to other divisions in Bangladesh. Uneducated mothers exhibited the largest percentage of stunting (44.3%), wasting (12.4%), and underweight (36.3%), while higher educated mothers found the lowest percentage of stunting (15.1%), wasting (6.2%), and underweight (10.9%). Underweighted mothers found the greater percentage of stunting (41.8%), wasting (13.7%), and underweight (33.2%). **Table 3** showed that age, residence, region, sex, mother education, father education, working, BMI, members, wealth, contraception, twin child, total children, birth order, birth interval, age at 1^st^ birth, vitamin A, water source, cooking fuel, toilet facility, and watching television were significantly associated with stunting; Mother education, BMI, child sex, BCG, vitamin A, and fever were significantly associated with wasting; Age, residence, region, sex, mother education, father education, working, BMI, members, wealth, breastfeeding, contraception, twin child, total children, birth order, birth interval, age at 1^st^ birth, fever, cough, water source, cooking fuel, toilet facility, and watching television were significantly associated with underweight (p-value<0.05).

**Table 3:** Background characteristics.

Predictors	Stunting n (%)	p-value	Wasting n (%)	p-value	Underweight n (%)	p-value
**Overall, n (%)**	2442 (31.3)		663 (8.5)		1762(22.5)	
**Age, n (%)**						
15–19	322(33.2)		89 (9.2)		215(22.0)	
20–24	820 (30.2)	0.048	222 (8.2)		559(20.5)	
25–29	679(31.0)		190 (8.7)	0.859	514(23.4)	<0.001
30–34	420 (32.1)		104 (8.0)		309(23.5)	
35–39	152(31.1)		45 (9.2)		121(24.5)	
40–44	39(34.5)		12 (10.6)		36(31.9)	
45–49	10(66.7)		1 (6.7)		8(53.3)	
**Residence, n (%)**						
Urban	710(26.7)		223 (8.4)	0.825	523(19.6)	<0.001
Rural	1732(33.7)	<0.001	440 (8.6)		1239(24.0)	
**Region, n (%)**						
Barisal	258 (31.4)		70 (8.6)		183(22.2)	<0.001
Chittagong	400 (32.1)		101 (8.1)		267(21.1)	
Dhaka	276 (25.0)		99 (9.0)		204(18.4)	
Khulna	206 (24.9)	<0.001	62 (7.5)	0.386	146(17.6)	
Mymensingh	330 (35.5)		87 (9.40)		248(26.5)	
Rajshahi	235 (29.0)		65 (8.1)		178(21.9)	
Rangpur	260 (28.9)		65 (7.2)		178(19.8)	
Sylhet	477 (41.2)		114 (9.90)		358(30.8)	
**Sex, n (%)**						
Male	2168 (31.7)	0.034	590 (8.7)	0.254	1582(23.0)	0.002
Female	274 (28.4)		73 (7.6)		180(18.6)	
**Mother education, n(%)**						
No education	247 (44.3)		69 (12.4)		203(36.3)	
Primary	894 (39.5)	<0.001	202 (8.9)	<0.001	639(28.1)	<0.001
Secondary	1106 (30.0)		312 (8.5)		778(21.0)	
Higher	195 (15.1)		80 (6.2)		142(10.9)	
**Father education, n(%)**					n: 7831	
No education	496 (43.4)		113 (9.9)		364(31.5)	<0.001
Primary	995 (37.5)	<0.001	230 (8.7)	0.190	700(26.3)	
Secondary	686 (27.7)		216 (8.7)		500(20.1)	
Higher	223 (15.9)		92 (6.6)		173(12.3)	
**Working, n(%)**						
No	1369 (29.3)	<0.001	392 (8.5)	0.782	992(21.3)	0.002
Yes	1083 (34.2)		271 (8.60)		770(24.3)	
**BMI, n (%)**						
Underweight	486 (41.8)	<0.001	159 (13.7)		387(33.2)	<0.001
Normal	1498 (32.4)		375 (8.1)		1055(22.6)	
Overweight	384 (23.8)		112 (6.9)		274(16.9)	
Obese	74 (19.1)		17 (4.40)	<0.001	46(11.9)	
**Members, n (%)**						
≤4	733 (29.6)	0.023	215 (8.70)		521(20.9)	0.025
>4	1909 (32.1)		448 (8.4)	0710	1241(23.2)	
**Religion, n (%)**						
Muslim	2235 (31.4)	0.741	610 (8.60)	0.543	1613(22.5)	0.791
Others	207 (30.8)		53 (7.90)		149(22.1)	
**Wealth, n (%)**						
Poorest	717 (41.0)		175 (10.0)		520(29.5)	
Poorer	611 (38.7)	<0.001	133 (8.5)	0.066	427(27.0)	
Middle	434 (30.8)		120 (8.5)		314(22.1)	<0.001
Richer	421 (27.2)		127 (8.2)		315(20.3)	
Richest	259 (17.1)		108 (7.2)		186(12.2)	
**Breastfeeding, n (%)**						
No	984 (31.7)	0.549	263 (8.5)		740(23.8)	0.028
Yes	1458 (31.1)		400 (8.6)	0.893	1022(21.6)	
**Decision contraception, n (%)**					n: 5379	
Mother	273 (37.2)		63 (8.6)		203(27.4)	
Husband	126 (35.7)	0.009	34 (9.6)	0.723	98(27.7)	0.008
Joint	1343 (31.6)		359 (8.5)		972(22.7)	
Other	4 (50.0)		0 (0.00)		3(37.5)	
**Twin child, n (%)**						
Single	2382 (31.1)	<0.001	648 (8.5)	0.368	1713(22.2)	<0.001
1^st^ of multiple	25 (41.7)		6 (10.0)		20(33.3)	
2^nd^ of multiple	35 (50.0)		9 (13.0)		29(41.4)	
**Child sex, n (%)**						
Male	1283 (31.5)	0.680	373 (9.2)	0.026	918(22.4)	0.870
Female	1159 (31.1)		290 (7.80)		944(22.6)	
**Total children, n (%)**						
≤ 2	1509 (28.6)	<0.001	442 (8.4)	0.587	1067(20.1)	<0.001
>2	933 (37.0)		221 (8.8)		695(27.4)	
**Birth order, n (%)**						
1	849 (28.6)		257 (8.7)		605(20.3)	<0.001
2–3	1184 (30.7)	<0.001	319 (8.3)	0.737	850(21.9)	
>3	409 (42.1)		87 (9.0)		307(31.5)	
**Birth interval, n (%)**					n: 4829	
<24	203 (39.2)		40 (7.8)		143(27.4)	<0.001
24–47	546 (37.1)	<0.001	135 (9.2)	0.456	398(26.9)	
>47	834 (29.6)		230 (8.2)		609(21.5)	
**Age at 1**^**st**^ **birth, n (%)**						
<18	1117 (34.2)	<0.001	286 (8.6)	0.538	820 (24.9)	<0.001
≥18	1325 (29.3)		377 (8.4)		942 (20.7)	
**BCG, n(%)**					n: 4843	
No	91 (26.8)		43 (13.0)	<0.001	79(23.1)	0.178
Yes	1414 (31.6)	0.062	351 (7.9)		903(20.1)	
**Vitamin A, n (%)**					n: 4843	
No	390 (27.3)		137 (9.7)	0.017	284(19.7	0.512
Yes	1115 (33.0)	<0.001	257 (7.6)		698(20.5)	
**Diarrhea, n (%)**					n: 7836	
No	2336 (31.6)	0.065	631 (8.5)	0.803	1674(22.5)	0.870
Yes	106 (27.1)		32 (8.2)		87(22.1)	
**Fever, n (%)**					n: 7836	
No	1602 (30.9)	0.243	395 (7.6)		1080(22.7)	<0.001
Yes	840 (32.2)		268 (10.3)	<0.001	681(25.9)	
**Cough, n (%)**					n: 7836	
No	1544 (31.4)	0.900	410 (8.4)		1074(21.7)	0.034
Yes	898 (31.2)		253 (8.8)	0.478	687(23.8)	
**Water source, n (%)**						
Improved	2049 (32.1)	0.002	550 (8.6)	0.458	260(18.3)	<0.001
Unimproved	393 (27.9)		113 (8.0)		1502 (23.4)	
**Cooking fuel, n (%)**					n: 7832	
Improved	292 (21.5)		112 (8.3)	0.782	1545(23.9)	<0.001
Unimproved	2148 (33.4)	<0.001	549 (8.5)		213(15.7)	
**Toilet facility, n (%)**						
Hygienic	1166 (35.6)	<0.001	297 (9.1)	0.120	855(25.9)	<0.001
Unhygienic	1276 (28.2)		366 (8.1)		907(20.0)	
**Watching television, n (%)**						
No	1119 (37.3)	<0.001	265 (8.9)	0.411	802(26.6)	<0.001
Yes	1323 (27.6)		398 (8.3)		960(19.9)	

### 3.2 Predictor’s selection by Boruta

**Fig 2:**
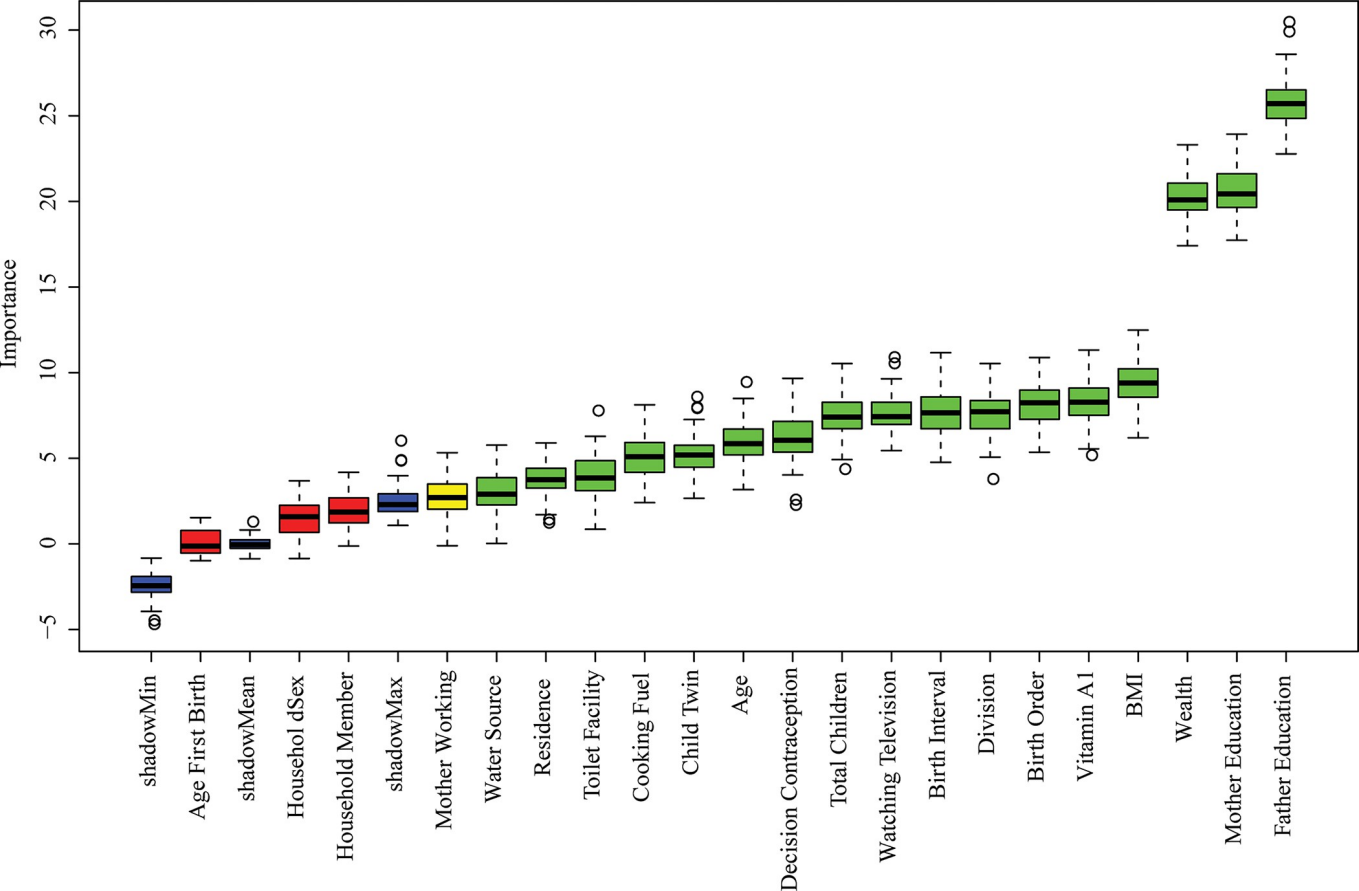
Stunting predictors selection using the Boruta method.

**Fig 3:**
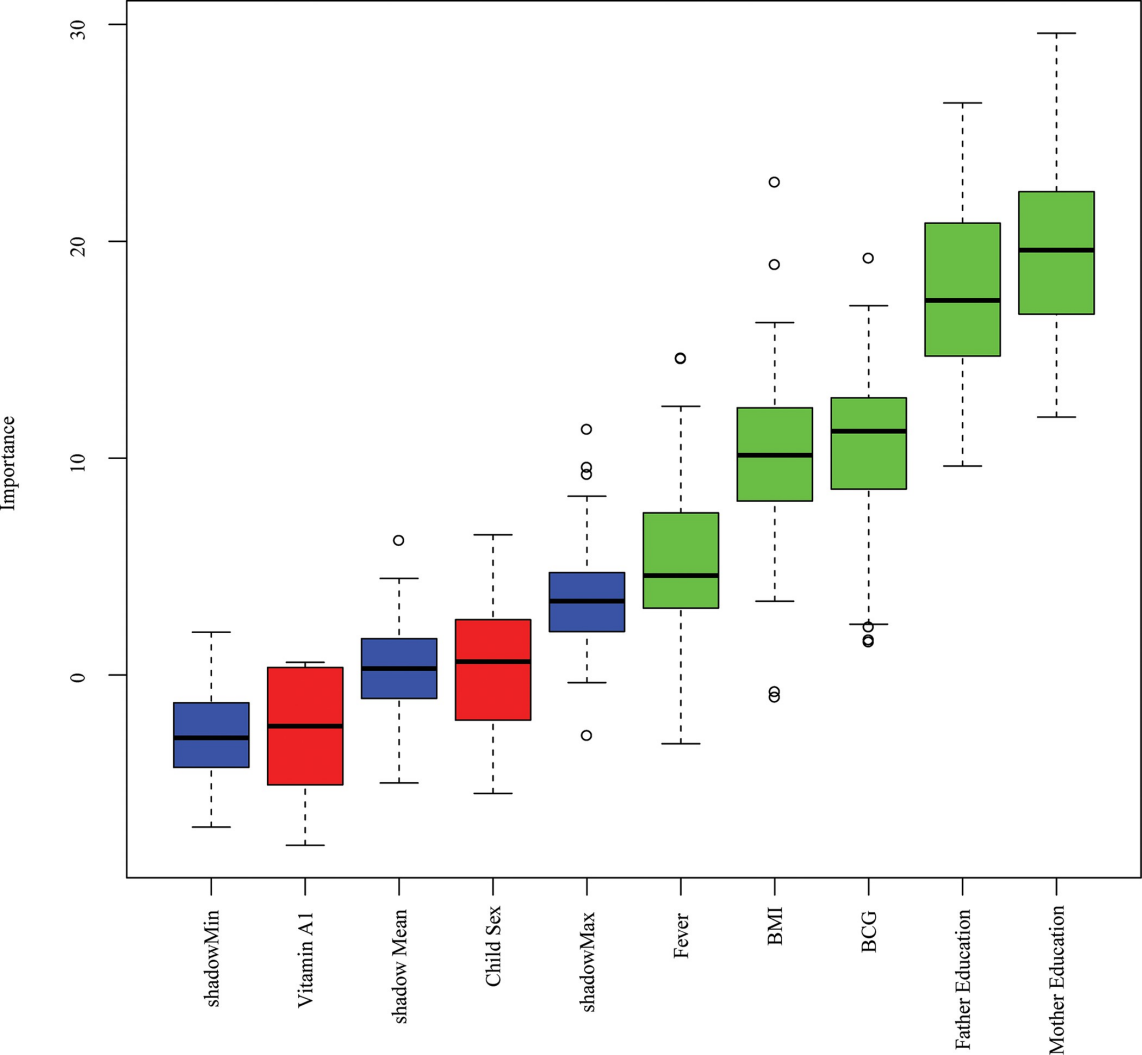
Wasting predictors selection using the Boruta method.

**Fig 4:**
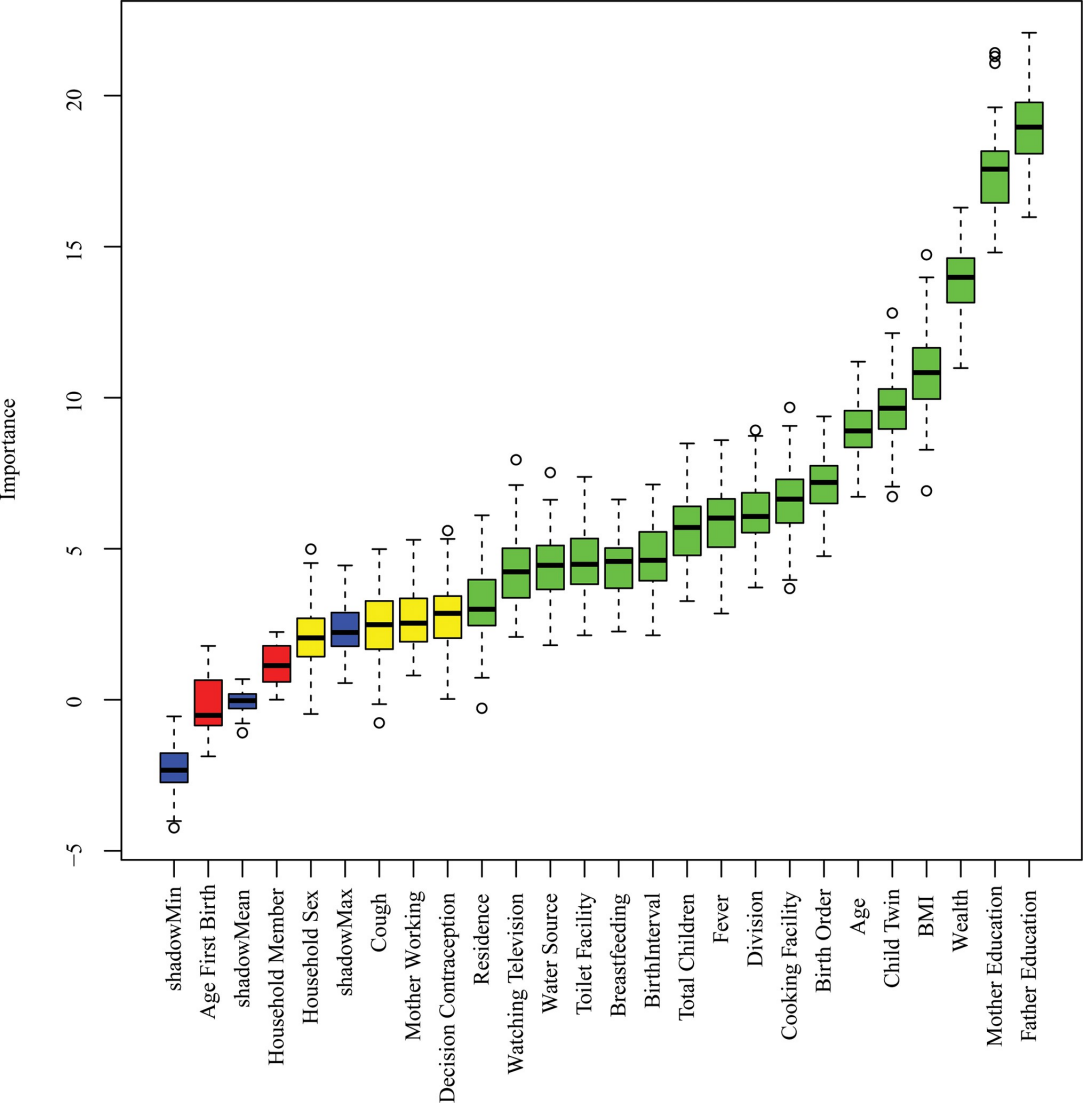
Underweight predictors selection using the Boruta method.

The predictors selection results based on Boruta for stunting, wasting, and underweight are displayed in **Figs 2–4**. The method revealed that there are 17 important predictors associated with stunting out of 21, 5 predictors for wasting out of 7, and 17 predictors for underweight out of 23. The selected predictors of stunting are water sources, residence, toilet facility, coking fuel, child twin, age, contraception, total children, watching television, birth interval, division, birth order, vitamin A, BMI, wealth, mother education, and father education (**Fig 2**); wasting are fever, BCG, BMI, father education, and mother education (**Fig 3**); underweight are residence, watching television, water source, toilet facility, contraception, birth interval, total children, fever, region, cooking facility, birth order, age, twin child, BMI, wealth, mother education, and father education (**Fig 4**). The selected predictors have been incorporated for predicting the risk of undernutrition (stunting, wasting, and underweight) among under-five children in Bangladesh.

### 3.3. Performance comparison of ML-based models

The predictive performance of four ML-based models is presented in **Table 4**.

**Table 4:** Performance (%) comparison of four models for stunting, wasting, and underweight.

Undernutrition	Models	Accuracy	Precision	Recall	F-score
		(%)	(%)	(%)	(%)
Stunting	LR	76.40	84.11	85.56	84.83
	ANN	80.49	86.42	87.32	86.87
	RF	79.92	87.01	85.88	86.44
	**XGB**	**81.73**	**88.28**	**89.41**	**88.84**
Wasting	LR	73.48	78.28	82.57	80.37
	ANN	75.82	82.17	84.44	83.29
	RF	74.53	80.16	83.07	81.59
	**XGB**	**76.15**	**83.35**	**85.45**	**84.39**
Underweight	LR	75.14	82.12	82.56	82.34
	ANN	78.44	83.43	84.32	83.87
	RF	77.52	82.41	85.68	84.01
	**XGB**	**79.13**	**87.27**	**87,53**	**87.40**

It is to be noticed that the XGB model attained the outperformed prediction accuracy of 81.73%, precision of 88.28%, recall of 89.41%, and F-score of 88.84% for stunting, while LR obtained the lowest accuracy of 76.40%, precision of 84.11%, recall of 85.56%, and F-score of 84.83%. The XGB model also demonstrated the highest level of predictive accuracy of 81.73%, precision of 88.28%, recall of 89.41%, and F-score of 88.84% for wasting. Furthermore, in comparison to the other models, the XGB model achieved an accuracy of 81.73%, a precision of 88.28%, a recall of 89.41%, and F-score of 88.84% for underweight.

**Fig 5:**
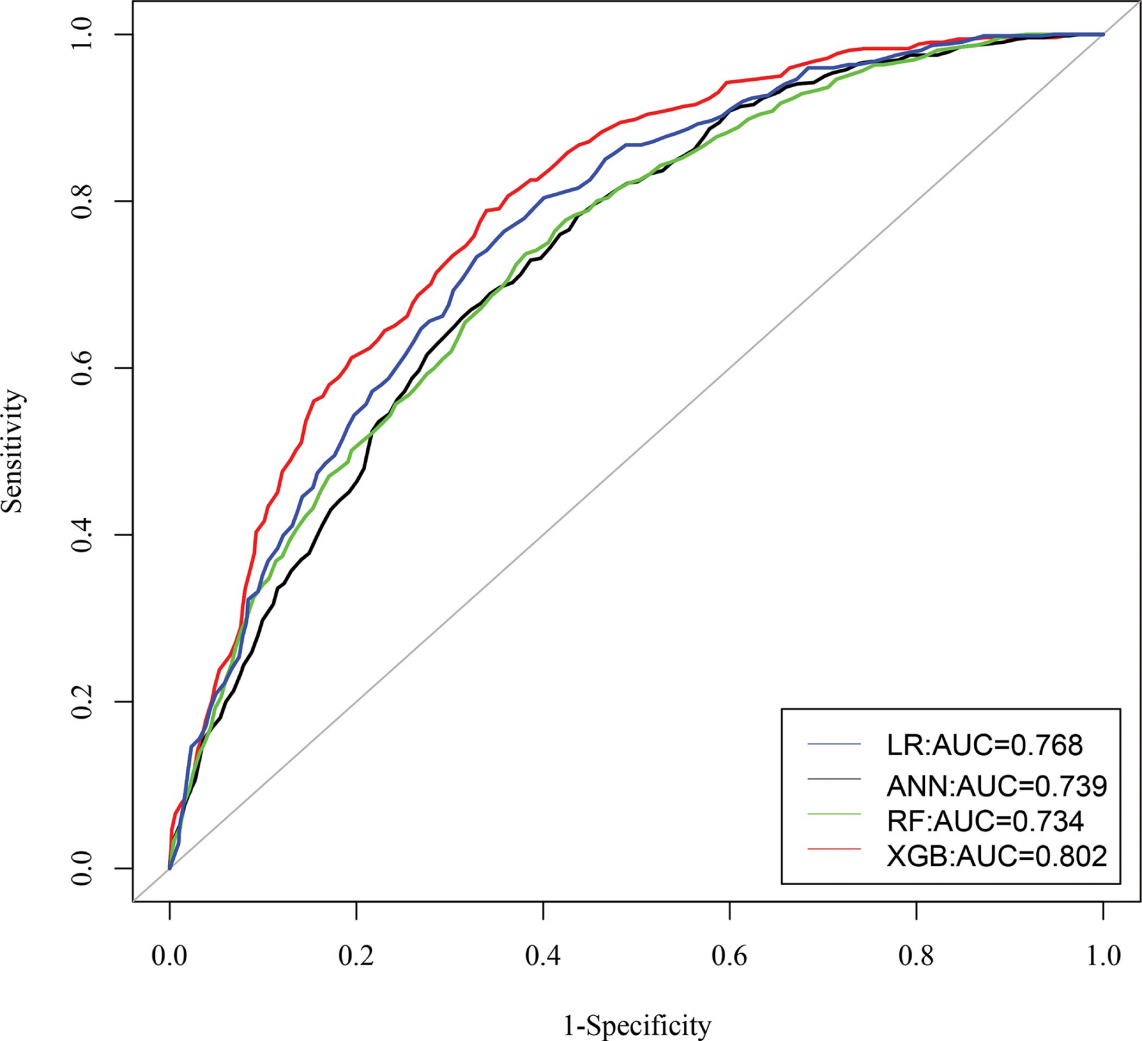
ROC curves of four models for stunting.

**Fig 6:**
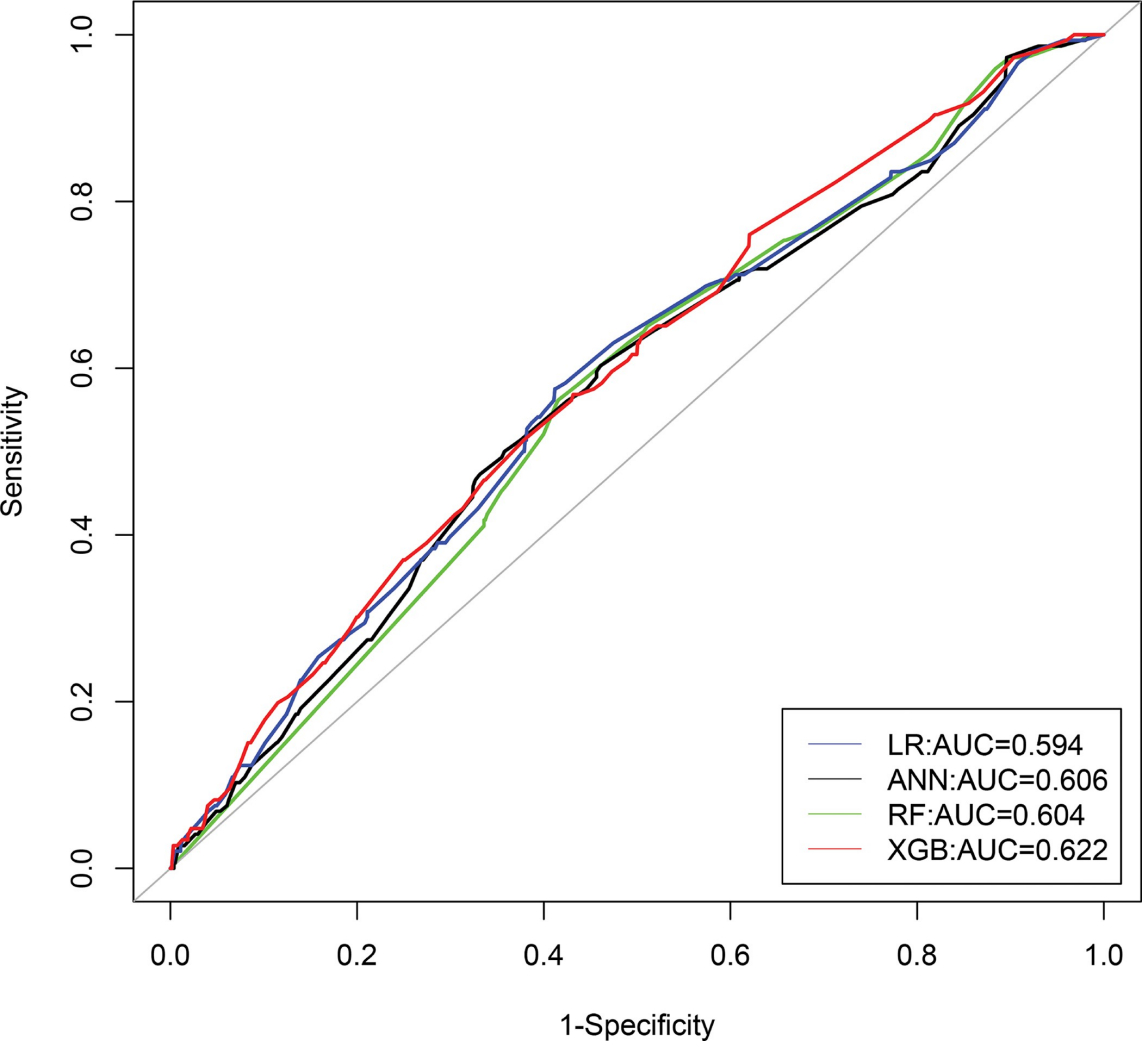
ROC curves of four models for wasting.

**Fig 7:**
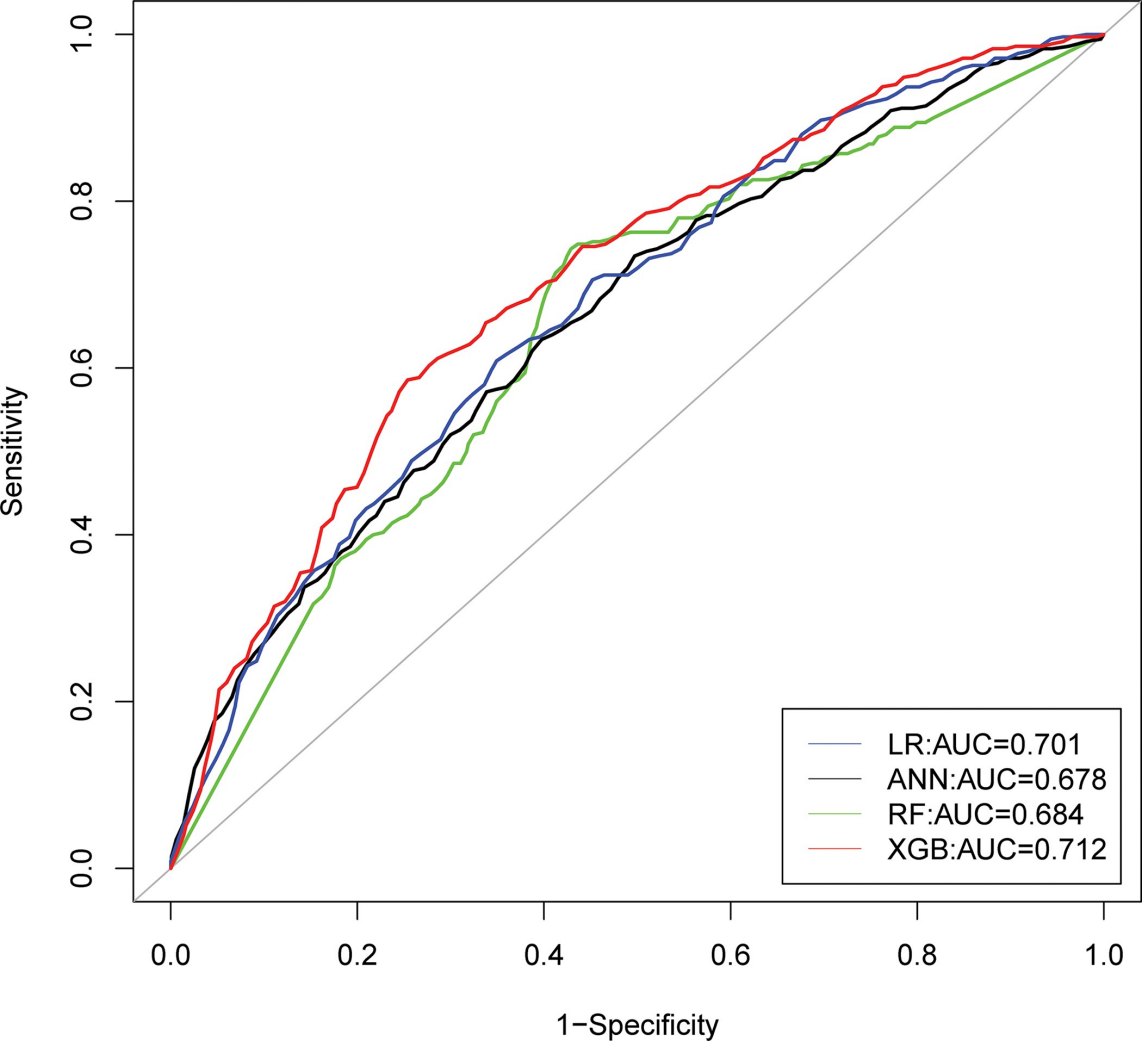
ROC curves of four models for underweight.

The corresponding ROC curves of stunting, wasting, and underweight was portrayed in **Figs 5–7**, and indicated that the XGB-based model acquired a larger area of ROC curve than other models: LR, ANN, and RF. Hence, the XGB-based model appears to be the most appropriate choice for predicting indicators of undernutrition among under-five children in Bangladesh.

### 3.4 Influencing predictors for undernutrition

To examine the importance of each predictor in the prediction, SHAP summary plot was made for the best XGB model by using SHAP values. In the SHAP summary plot, the x-axis represents the SHAP values, while the y-axis represents the contribution of each predictor. A predictor with a higher SHAP value is more likely to influence the occurrence of undernutrition. The red dots indicate higher values, while the blue dots indicate lower values. SHAP summary plot of the XGB model for stunting, wasting, and underweight was depicted in (**S1–S3 Figs**). The SHAP methods revealed that father education, wealth, mother education, BMI, birth interval, vitamin A, watching television, toilet facility, residence, and water source possess a higher SHAP value exceeding zero, thereby indicating that they are the influential predictors of stunting (**S1 Fig**). While, BMI, mother education, and BCG (**S2 Fig**) are influential predictors of wasting; and father education, wealth, mother education, BMI, birth interval, toilet facility, breastfeeding, birth order, and residence (**S3 Fig**) are the influential predictors of underweight.

## 4. Discussion

Nutrition is crucial for maintaining good health and promoting the growth and well-being of the human body at every stage of life. Severe malnutrition can lead to life-threatening consequences such as inhibited growth, impaired immune systems, and even death. Thus, this study highlighted the usefulness of several ML algorithms utilizing the most recent BDHS, 2017–2018 data to explore an appropriate explainable model that predicts the risk of undernutrition among children under five and determines the predictors that influence it. For each undernutrition indicator, four widely used ML-based algorithms were trained using the important predictors obtained by the Boruta method. The models’ performance was evaluated through accuracy, precision, recall, F-score, and ROC curve with AUC value. Based on the performance metrics, the XGB-based model was found superior to others for predicting the risk of undernutrition. The latest study conducted by Anku in Ghana, demonstrated that the XGB model was the best performer in predicting undernutrition among under-five children [51]. Other investigations also reported that the XGB-based model was the most precise for predicting undernutrition among children under five [52, 53]. The superiority of the XGB model may be due to its operation within the gradient boosting framework, which sequentially adds weak learners (typically DTs) and iteratively corrects errors by the preceding weak learners to achieve accurate prediction and it has the capability to effectively handle high-dimensional and complex data for classification [25]. However, the SHAP method in the XGB-based model reveals that the predictors of undernutrition vary across the three different indicators. Nevertheless, mother education and BMI are the coexistent predictors across three indicators of stunting, wasting, and underweight. This result is in line with the most recent research carried out in different nations [14, 54–57]. A mother who has received education may have a better awareness of the nutritional needs of her children. Better child feeding techniques, such as introducing supplementary foods to infants on time and exclusively breastfeeding during the first six months of a newborn’s life, are strongly linked to a decreased incidence of undernutrition in children [52]. Furthermore, mothers with higher levels of education are more likely to employ family planning [16], to use resources for the family effectively [19, 53], and to improve their children’s access to healthcare [25, 53]. The growth and development of a child greatly depend on the nutritional status of his/her mother. Mothers who are underweight face a much greater risk of stunting and wasting in comparison to mothers who have a normal weight [58]. Children of mothers with normal or above BMI have a lower risk of being underweight. Therefore, policymakers should prioritize the nutritional status of children to reduce malnutrition among them effectively. The coexistent predictors of stunting and underweight are the father’s education, wealth, birth interval, toilet facility, and residence. The socioeconomic status of the family influences the growth and development of the child, as well as their access to food security. Children from low-income families have more difficulty accessing food and medical care, which increases their risk of illness and death. This study demonstrated that the risk of stunting and underweight was highest in the poorest households, which coincided with recent research from neighboring countries including Bangladesh [56, 59, 60]. Birth spacing also has an impact on the nutritional status of under-five children. A lengthy gap between births is beneficial for the health and nutrition of both mothers and children, which was corroborated with the previous studies [61, 62]. Children living in rural areas with poor sanitation are more likely to experience stunting and being underweight. Improving access to clean and safe toilet facilities, along with promoting proper sanitation and hygiene practices, is essential for preventing childhood undernutrition and promoting overall health and well-being. Additionally, vitamin A, watching television, and water sources are also the influencing predictors of stunting, BCG of wasting, breastfeeding, and birth order of underweight. These findings are aligned with the earlier studies [63, 64]. Insufficient levels of vitamin A can impact various elements of growth and maturation, such as cellular growth, immune response, skeletal development, and hormonal equilibrium, all of which play a key role in the hindered growth of children [65]. The first-born siblings were prone to nurturing a deep sense of responsibility, the middle siblings a hunger for attention, and the youngest siblings a thirst for adventure and rebellion [66].

## 5. Conclusion

This study utilized four different ML-based algorithms to explore an appropriate explainable predictive model for the prediction of undernutrition among under-five Bangladeshi children. The comprehensive findings from our experiments indicate that, out of the four models, the XGB model is the most appropriate for predicting children with undernutrition. The SHAP method reveals that father education, wealth, mother education, BMI, birth interval, vitamin A, watching television, toilet facility, residence, and water source are the influential predictors of stunting among under-five children in Bangladesh. While, BMI, mother education, and BCG of wasting; and father education, wealth, mother education, BMI, birth interval, toilet facility, breastfeeding, birth order, and residence of underweight. The proposed integrating framework may be used to create an automated tool in clinical settings that correctly detect children who are undernourished in their early stages. With the help of this information, a healthcare provider can make proper decisions and formulate patient-specific treatment plans to mitigate wait times and healthcare expenses. Ultimately, our research may greatly enhance the care of undernourished children and assist decision-makers in taking appropriate initiatives to fulfill the Sustainable Development Goal (SDG) of decreasing pediatric undernutrition in Bangladesh by 2030.

### 5.1. Limitations of the study

This study is cross-sectional in nature, thereby restricting our capacity to establish causal relationships. While investigating several plausible factors, the data was absent in some other significant predictors, such as poor consumption of vitamin supplements, not up-to-date immunization, and so on. The important predictors of undernutrition will aid in obtaining precise results and enhanced model interpretability.

## Supporting information

S1 Fig SHAP summary plot of the XGB model for stunting. (TIF)

S2 Fig SHAP summary plot of the XGB model for wasting. (TIF)

S3 Fig SHAP summary plot of the XGB model for underweight. (TIF)
