# Supplementary figures and images for: Prediction of undernutrition and identification of its influencing predictors among under-five children in Bangladesh using explainable machine learning algorithms
Source: PLoS One. 2024 Dec 6;19(12):e0315393. doi: 10.1371/journal.pone.0315393 (PMC11623790; doi:10.1371/journal.pone.0315393)

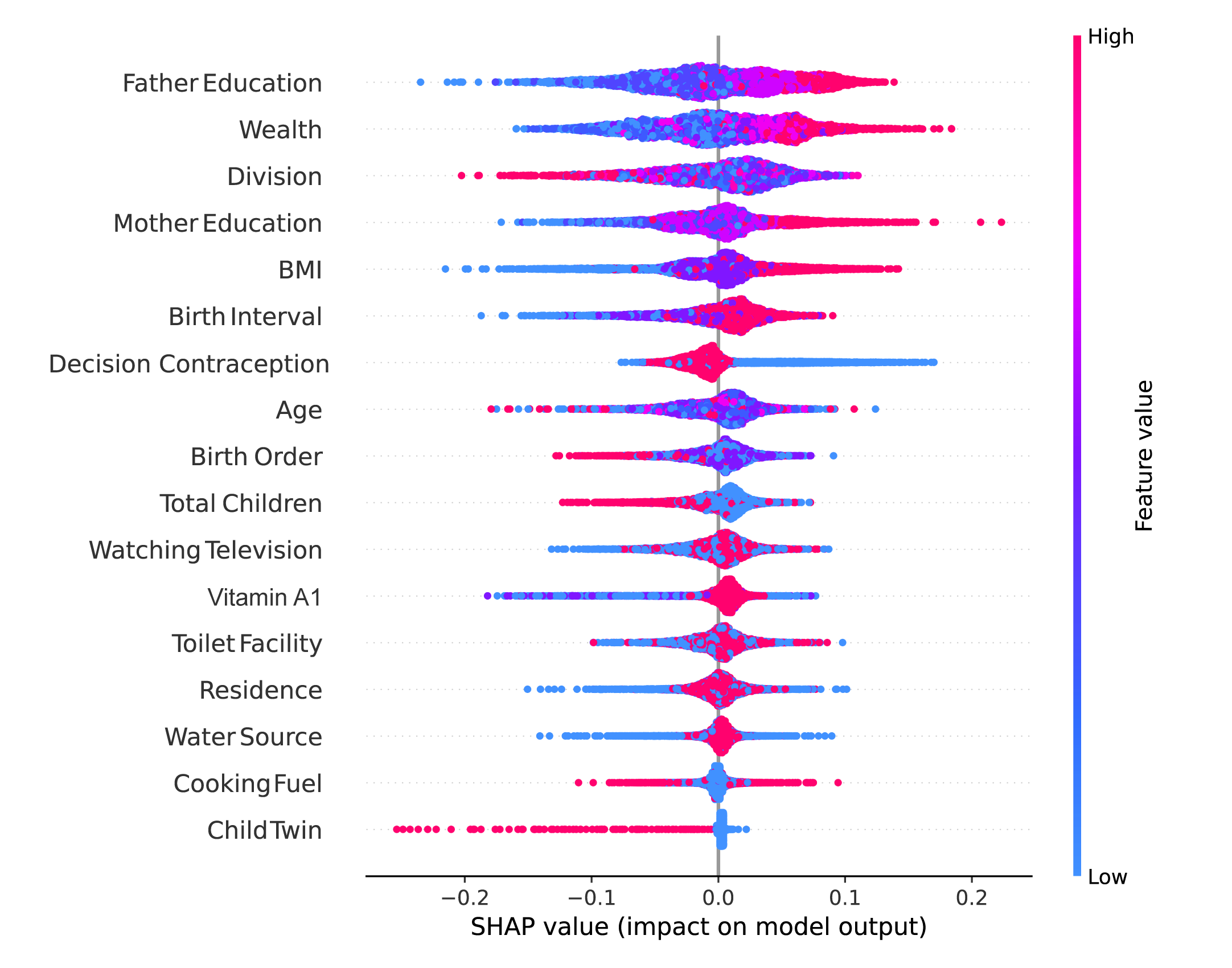

Supplement: S1 Fig — (TIF) [file pone.0315393.s001.tif]

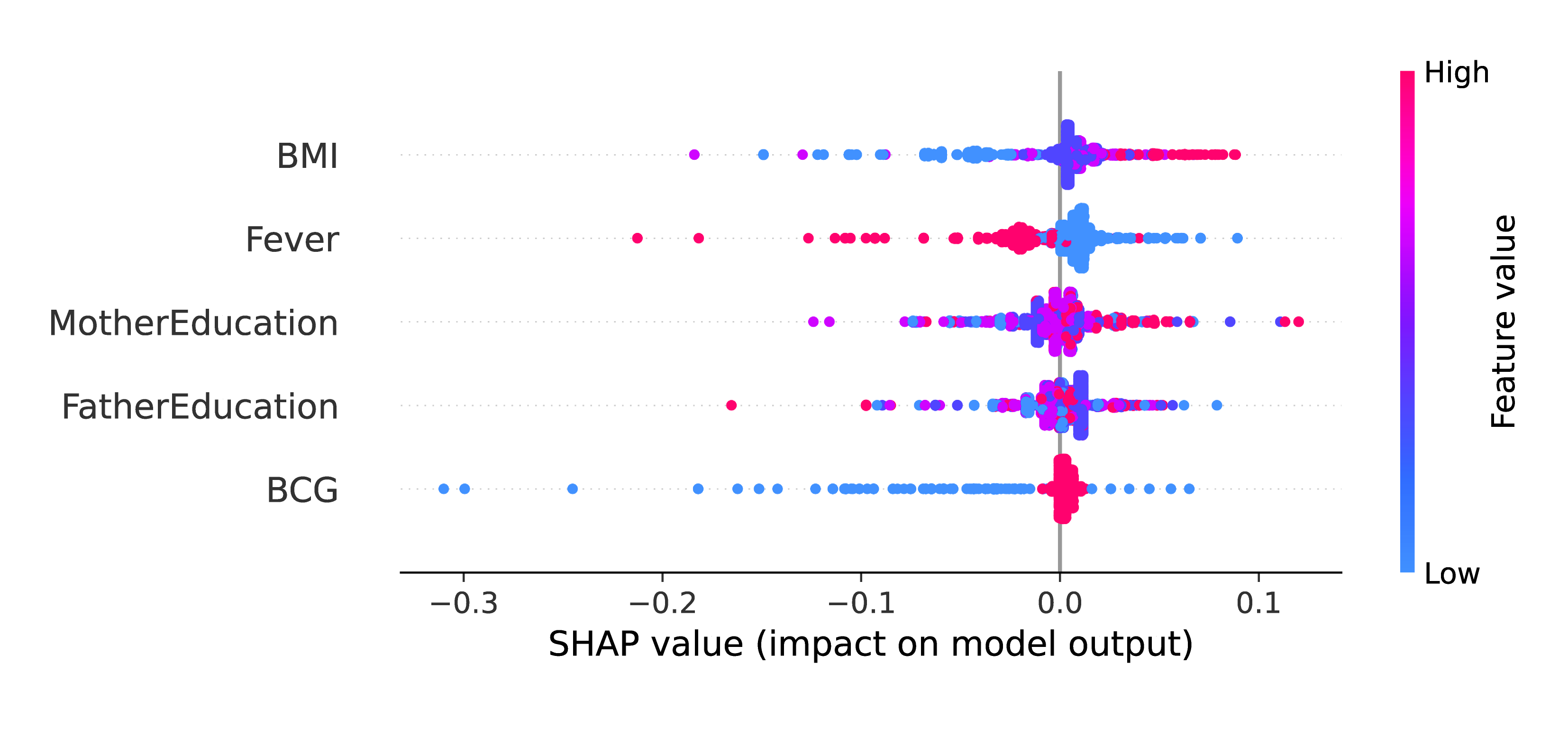

Supplement: S2 Fig — (TIF) [file pone.0315393.s002.tif]

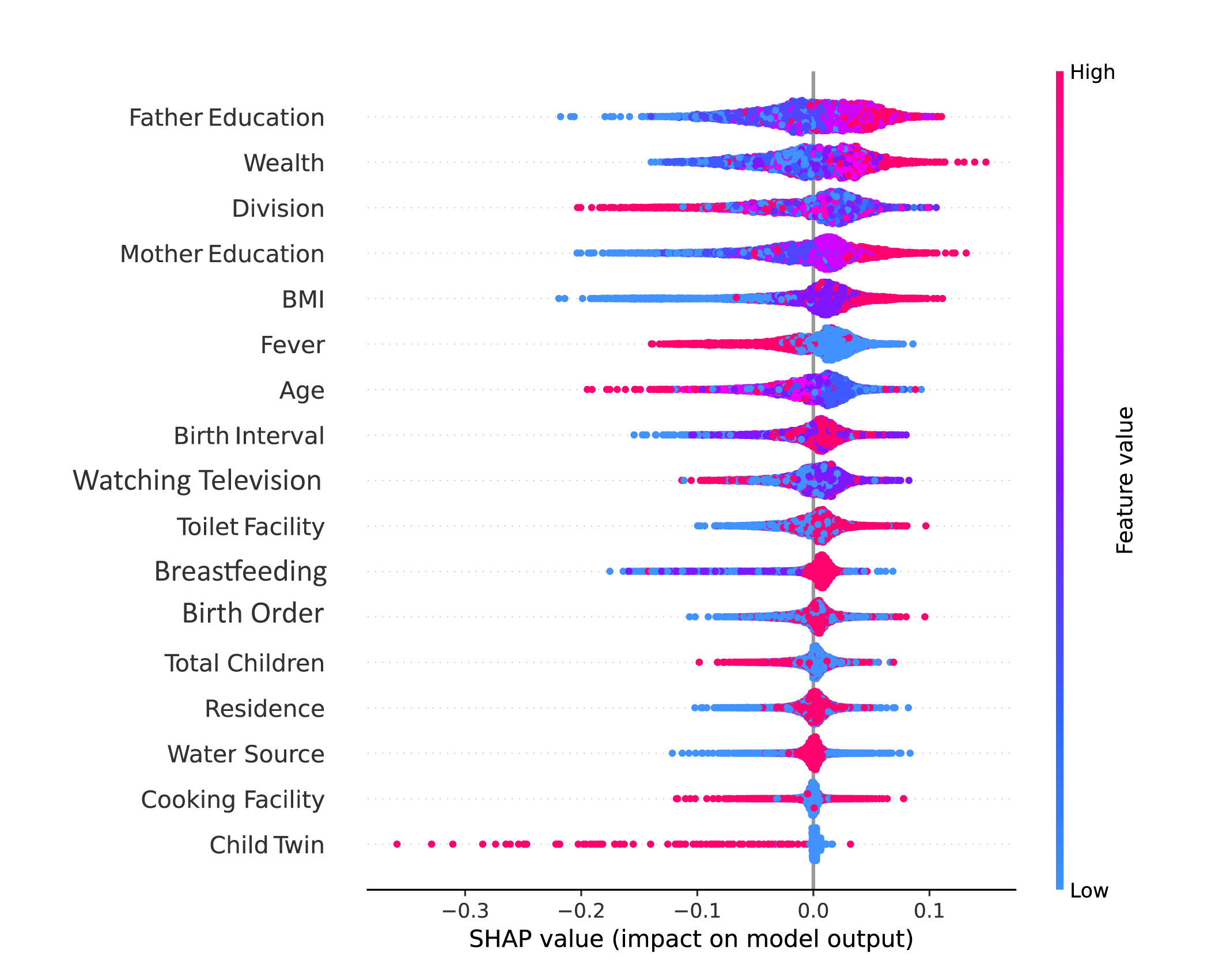

Supplement: S3 Fig — (TIF) [file pone.0315393.s003.tif]
